# Chronic stress and coping mechanisms among nurses in Lango sub‐region, northern Uganda

**DOI:** 10.1002/nop2.1831

**Published:** 2023-05-18

**Authors:** Amir Kabunga, Eustes Kigongo, Samson Udho, Ann Grace Auma, Ponsiano Okalo, Brenda Apili, Namata Halimah, Viola Nalwoga

**Affiliations:** ^1^ Department of Psychiatry, Faculty of Medicine Lira University Lira Uganda; ^2^ Department of Environmental Health and Disease Control Lira University Lira Uganda; ^3^ Department of Nursing and Midwifery, Faculty of Nursing and Midwifery Lira University Lira Uganda; ^4^ Department of Mental Health Makerere University Kampala Uganda

**Keywords:** chronic stress, coping strategies, nurses, stress

## Abstract

**Aim:**

This study aimed to assess chronic stress and coping mechanisms among nurses in Lango sub‐region, northern Uganda, conducted between May and June 2022.

**Design:**

Institutional‐based cross‐sectional design conducted between May and June 2022.

**Methods:**

The study included 498 participants recruited from six health facilities. A 12‐Item Short Form Survey tool was used to collect data on chronic stress, while a researcher‐developed questionnaire was used to collect data on coping strategies. Descriptive statistics, binary logistic regression and multiple regression were conducted for data analysis. A *p*‐value of 0.05 was considered statistically significant.

**Results:**

Out of 498 participants, 153 (30.7%) were aged between 31 and 40 years, 341 (68.5%) were female, 288 (57.8%) were married, and 266 (53.4%) had less than Diploma. Of the 498 participants, 351 (70.5%) experienced chronic stress. The protective factors against chronic stress were being married (AOR: 0.132; 95% CI: 0.043–0.408; *p* < 0.001), optimizing shift length (AOR: 0.056; 95% CI: 0.027–0.115; *p* < 0.001), religiosity/Spirituality (AOR: 2.750; 95% CI: 1.376–5.497; *p* = 0.004), and regular exercise and breaks (AOR: 0.405; 95% CI: 0.223–0.737; *p* = 0.003).

## 1 BACKGROUND

Nurses in the twenty‐first century encounter difficulties including, a lack of nurses, poor efficacy, workload issues, system change and an increase in the complexity of clinical procedures (Betke et al., 2021). It is now widely acknowledged as a serious public health problem that social problems and stressful life events may have a negative influence on nurses' health and well‐being (Gavin et al., 2020). It is evident that there is a nursing shortage in Sub‐Saharan Africa, and this could lead to serious healthcare issues (Ayalew et al., 2021), and this shortage is associated with chronic stress among nurses (Heuel et al., 2022; Wollesen et al., 2019). Numerous studies have examined the strain that chronic stress causes on medical professionals (Heuel et al., 2022; Kersting et al., 2019; Wollesen et al., 2019). The majority of research, however, comes from high‐income nations and focuses on stress among hospital staff members rather than nurses working in low‐resource environments.

Chronic stress is thought to be the result of extended exposure to one or more acute stressors that are repeated or frequent (Ceccato et al., 2018). Chronic stress can result in burnout symptoms, job turnover and absenteeism (Heuel et al., 2022). Also, chronic stress raises the likelihood of poor health, and it has been demonstrated that poor nurses' health is linked to poor patient outcomes and a higher frequency of medical errors (Babapour et al., 2022; Bahrami, 2016). Thus, methods for reducing chronic stress among nurses could enhance patient care. There is a need to improve the well‐being of the current nursing by reducing stress, which could enhance patient care.

The authors of the transactional stress theory, Richard Lazarus and Susan Folkman, claim that coping with stress is a constantly changing cognitive and behavioural effort that is concentrated on particular internal and external struggles in circumstances that are perceived as burdensome or beyond one's capacity. According to this theory, when a person interprets environmental stresses, stress may be caused depending on their contextual and personal resources (Lazarus & Folkman, 1987). As a result, different people may have diverse stress responses when exposed to the same objective stressor (Jacoby et al., 2021). Environmental resources include organizational traits including employment stability and control, participation possibilities, feedback and support from co‐workers and supervisors (López‐Cabarcos et al., 2022). Personal resources reflect a person's personality, views, knowledge and behavioural patterns (Lim et al., 2010). In line with transactional theory, environmental and personal resources may offer protection against the negative effects of stress on one's health (Garrosa et al., 2011).

Nurses in Uganda are under stress as a result of the country's statistically significant human resource shortages (Shiden et al., 2017). When compared to the WHO's recommended ratios of 1:2 for complicated illnesses and 1:5 for common illnesses, the Ugandan situation—one nurse/midwife to a population of 3065 patients—is highly difficult and stressful (Musiimenta et al., 2022). As a result, nurses in Uganda frequently complain about being overworked (Musiimenta et al., 2022). This is demonstrated by the low staff retention rate, which is made worse by the fact that many choose to quit the field without any support and infectious diseases, especially COVID‐19 (Nadeem et al., 2021). Chronic stress is made worse for these nurses by the fact that they work amidst few resources (Nabirye et al., 2011). Thus, the present study assessed the prevalence and coping mechanisms among nurses in Lango sub‐region, northern Uganda.

## 2 METHODS

### 2.1 Study participants, design and setting

This study was conducted among 550 randomly selected nurses working in six health facilities. All nurses who were present during data collection were included in the study. Also, the participants had to be Registered Nurses working in the Lango sub‐region, in northern Uganda. There are currently nine districts in the Lango sub‐region: Alebtong, Amolatar, Apac, Dokolo, Kole, Lira, Oyam, Otuke and Kwania District. This institutional‐based cross‐sectional research design was applied to examine the prevalence of chronic stress and nurses' coping mechanisms. The records of nurses in each hospital were used to identify potential study participants. The participants were selected using a simple random sampling method.

### 2.2 Study instruments

The questionnaire tools included demographic, SF‐12 is a 12‐item and coping‐related items. The SF‐12 is a 12‐item scale that assesses five different chronic stress‐related experiences: chronic anxiety, work‐related overload, social overload, overextending oneself and lack of recognition. A 5‐point Likert scale, from 0 (never) to 4, was used to gauge how frequently respondents had relevant experience in the previous 3 months (very often). The total SF‐12 scores are calculated by adding the results of all the items. The SF‐12 total scores ranged from 0 to 48, and higher values imply higher levels of chronic stress. Respondents' scores are divided into two groups: those who are not chronically stressed (score ≤ 15) and those who are chronically stressed (scored > 15). Good discriminant validity, content validity, internal consistency and test–retest reliability have all been demonstrated for the SF‐12 (Shah & Brown, 2020). The Cronbach's alpha for SF‐12 in this study was 0.92. The first part of the questionnaire included a section relating to the demographics of the respondents. Finally, coping strategies employed by nurses were captured using researcher‐formulated items. The tool was pilot‐tested with 40 nurses in a local hospital. Reliability was assessed using Cronbach's alpha, and it showed *α* > 0.80.

### 2.3 Procedure

The study was conducted between May and June 2022. In the Lango sub‐region of northern Uganda, the first contact was made with district health and hospital administration officials. Potential participants were located after the research protocol was approved. The risks and benefits were discussed with the participants before participation in the study. They were then told what the study was about, given the option to voluntarily withdraw if necessary and asked to take part after signing the written consent forms. Completing the questionnaire took about 20 min.

### 2.4 Data analysis

Data were entered into excel before being exported into SPSS version 25 for analysis. Descriptive statistics including frequencies, percentages and cross‐tabulations were used to describe the study population's relevant variables. Binary logistic regression analysis was performed to assess the association between the independent and dependent variables. The odds ratio of 95% CI was used to determine the direction and strength of the association. Multiple regression analysis was conducted and the aOR and 95% CI were estimated to identify the factors associated with chronic stress. A *p*‐value of <0.05 was considered statistically significant.

## 3 RESULTS

### 3.1 Sociodemographic characteristics of the participants (*n* = 498)

Five hundred fifty nurses in total were selected to take part in the study, and 498 (90.5%) of them consented to fill out the questionnaires. Our final analysis sample in Table 1 included 498 participants, giving a 90.5% response rate. Out of 498 participants, 153 (30.7%) were aged between 31 and 40 years, 341 (68.5%) were female, 288 (57.8%) were married, and 266 (53.4%) had less than Diploma. Of the 498 participants, 351 (70.5%) experienced chronic stress (Table 1) and it was associated with marital status (*p* < 0.001).

**TABLE 1:** Sociodemographic characteristics of the participants (*n* = 498).

Explanatory variables	Category	Frequency	Chronic stress		*p*‐Value
			Absent 147 (29.5)	Present 351 (70.5%)	
Age	20–30 years	139 (27.9%)	46 (33.1%)	93 (66.9%)	0.279
	31–40 years	153 (30.7%)	50 (32.7%)	103 (67.3%)	
	41–50 years	102 (20.5%)	25 (24.5%)	77 (75.5%)	
	>50 and above	104 (20.9%)	26 (25.0%)	78 (75.0%)	
Gender	Male	157 (31.5%)	54 (34.4%)	103 (65.6%)	0.105
	Female	341 (68.5%)	93 (27.3%)	248 (72.7%)	
Marital status	Single	45 (9.0%)	5 (11.1%)	40 (88.9%)	<0.001*
	Married	288 (57.8%)	131 (45.5%)	153 (54.5%)	
	Divorced/Separated	75 (15.1)	6 (8.0%)	69 (92.0%)	
	Widowed	90 (18.1%)	5 (5.6%)	85 (94.4%)	
Education levels	Less than Diploma	266 (53.4%)	75 (28.2%)	191 (71.8%)	0.096
	Diploma	161 (32.3%)	48 (29.8%)	113 (70.2%)	
	Bachelor's degree	53 (10.6%)	14 (26.4%)	39 (73.6%)	
	Postgraduate	18 (3.6%)	10 (55.6%)	8 (44.4%)	

* Statistically significant variable at *p* < 0.05.

### 3.2 Chronic stress and associated factors (*n* = 638)

Binary logistic regression analysis (Table 2) was conducted to identify potential factors associated with chronic stress. Association was observed between the elevated levels of chronic stress, optimizing shift length (*p* < 0.001), religiosity/Spirituality (*p* = 0.006), regular exercise and breaks (*p* < 0.001) and staff autonomy and decision‐making (*p* = 0.012).

**TABLE 2:** Chronic stress and associated factors (*n* = 498).

Explanatory variables	Frequency	Chronic stress		*p*‐Value
		Absent 153 (24.0)	Present 351 (76.0%)	
Optimize shift length				
No	256 (51.4%)	134 (52.3%)	122 (47.7%)	<0.001*
Yes	242 (48.6%)	13 (5.4.03%)	229 (94.6%)	
Religiosity/Spirituality				
No	126 (25.3%)	25 (19.8%)	101 (80.2%)	0.006*
Yes	372 (74.7%)	122 (32.8%)	250 (67.2%)	
Regular exercise and breaks				
No	279 (56.0%)	106 (38.0%)	173 (62.0%)	<0.001*
Yes	219 (44.0%)	41 (18.3%)	178 (81.3%)	
Establishing boundaries				
No	67 (13.5%)	24 (35.8%)	43 (64.2%)	0.224
Yes	431 (86.5%)	123 (28.5%)	308 (71.5%)	
Staff autonomy and decision‐making				
No	79 (15.9%)	14 (17.7%)	65 (82.3%)	0.012*
Yes	419 (84.1%)	133 (31.7%)	286 (68.3%)	
Provide training in conflict resolution				
No	94 (18.9%)	34 (36.2%)	60 (63.8%)	0.116
Yes	404 (81.1%)	113 (28.0%)	291 (72.0%)	

* Statistically significant variable at *p* < 0.05.

### 3.3 Multivariate analysis (*n* = 498)

The results in Table 3 indicate that the correlates of chronic stress among participants were being a widow (aOR: 0.132; 95% CI: 0.043–0.408; *p* < 0.001), failure to optimize shift length (aOR: 0.056; 95% CI: 0.027–0.115; *p* < 0.001), low religiosity/Spirituality (aOR: 2.750; 95% CI: 1.376–5.497; *p* = 0.004) and lack of regular exercise and breaks (aOR: 0.405; 95% CI: 0.223–0.737; *p* = 0.003).

**TABLE 3:** Multivariate analysis (*n* = 498).

Variables	Frequency	COR 95% CI	AOR 95% CI	*p*‐Value
Marital status				
Single	139 (27.9%)	0.471 (0.129–1.719)	1.576 (0.355–6.966)	0.550
Married	153 (30.7%)	0.070 (0.028–0.179)	0.132 (0.043–0.408)	<0.001*
Divorced/Separated	102 (20.5%)	0.676 (0.198–2.311)	0.914 (0.215–3.893)	0.0903
Widowed	104 (20.9%)	1	1	
Optimize shift length				
No	256 (51.4%)	0.052 (0.028–0.095)	0.056 (0.027–0.115)	<0.001*
Yes	242 (48.6%)	1	1	
Religiosity/Spirituality				
No	126 (25.3%)	1.972 (1.210–3.213)	2.750 (1.376–5.497)	0.004*
Yes	372 (74.7%)	1	1	
Regular exercise and breaks				
No	279 (56.0%)	0.376 (0248–0.570)	0.405 (0.223–0.737)	0.003*
Yes	219 (44.0%)	1	1	

*Note*: 1: Reference category.

* Statistically significant variable at *p* < 0.05.

## 4 DISCUSSION

The study aimed to examine the prevalence and Coping with chronic stress among nurses in Uganda. Our results indicated that, of the 498 participants, 351 (70.5%) experienced chronic stress. Our results show that the correlates of chronic stress among participants were being a widow (aOR: 0.132; 95% CI: 0.043–0.408; *p* < 0.001), failure to optimize shift length (aOR: 0.056; 95% CI: 0.027–0.115; *p* < 0.001), low religiosity/Spirituality (aOR: 2.750; 95% CI: 1.376–5.497; *p* = 0.004), and lack of regular exercise and breaks (aOR: 0.405; 95% CI: 0.223–0.737; *p* = 0.003). Past studies with nurses indicated high‐stress levels (Heuel et al., 2022). Our results are favourably in tandem with the findings of Heue and colleagues (Heuel et al., 2022), which showed that 84% of the nurses were chronically stressed. This result, however, demonstrates the need for developing interventions for nurses throughout the nation.

As we had anticipated, our findings demonstrated that married nurses experienced less chronic stress than single, divorced or separated. This means that marriage acts as a protection against chronic stress. It is believed that those in marriage have better mental health because they offer more social support, financial assistance and a greater sense of purpose in life (Clark et al., 2020). Findings from a study conducted in five Asian nations showed a link between poor well‐being and not having someone with whom to discuss statistically significant issues (Yamaoka, 2008). Marriage establishes connections between persons, social groups like extended families and other social institutions that provide extra social benefits (Perelli‐Harris et al., 2018). In line with our findings, a study showed that widowhood was consistently linked to poor mental health (Grundström et al., 2021).

The findings of this study demonstrate that nurses who turned to religion as a coping mechanism were likely to have low chronic levels of stress. Nursing professionals frequently deal with a range of stressors, and it has been shown that they use religious or spiritual coping mechanisms to lessen the harmful effects of work stress (Perera et al., 2018). Spirituality/religiosity may offer resources that faster coping with stressful events. According to a prior study, spirituality/religiosity can inspire both self‐forgiveness and divine forgiveness, both of which are favourably correlated with psychosocial well‐being (Long et al., 2020). In line with our findings, most research shows that higher spirituality/religiosity is associated with fewer mental health issues (De Diego‐Cordero et al., 2021). Thus, various medical and nursing organizations' policies and policy statements should embrace spirituality/religiosity.

Our results revealed that optimal shift length was associated with low chronic stress. Long‐shift nurses are also more likely than their counterparts to report mental health problems and plan to exit the profession (Santana et al., 2020). Stress may emerge from modifications to the skill mix brought on by the adoption of 12‐h shifts (Ejebu et al., 2021). Although shift work in this study is associated with less chronic stress, there has been conflicting evidence associating shift work with mental health (Sweeney et al., 2021). Studies show that shift work and poor mental health were strongly associated (Li et al., 2022). Additionally, shift work was linked to a 33% higher incidence of depressive symptoms, according to research by Rosenberg et al., poor mental health conditions were more prevalent in shift employees than in non‐shift workers (Rosenberg, 2020). In line with our findings research using sizable samples has linked shift work and mental health (Sweeney et al., 2021). These contradicting findings may be explained by regional variations in the features of the workplace, the economy and shifts in employment.

The findings of this study demonstrate a direct relationship between chronic stress and regular exercise and breaks with those health workers who have regular exercise and breaks during work being more likely to experience less chronic stress compared with their counterparts who do not have exercise and breaks. This means regular exercise and taking breaks at work are crucial for overall health. Therefore, workplace exercises improve workers' performance and quality of life (Martinez, 2021). The impact of stress can be lessened and prevented in large part by engaging in physical activity (Odigie, 2016).

### 4.1 Limitations

This present study had its limitations. The self‐reporting method of data collection may have led to some degree of social desirability bias. Also, casual interpretation of the findings is speculative due to the nature of the cross‐sectional study design. Data collection was limited to participants in the Lango sub‐region of northern Uganda, which may limit the generalization of the results. Additionally, it was not mentioned if the nurses completed the surveys at work or in their free time. Nonetheless, the sample size was relatively big.

## 5 CONCLUSION

The chronic stress level in nurses was high and protective factors were being married, optimizing shift length religiosity/Spirituality and regular exercise and breaks.

### 5.1 Relevancy for clinical practice

These results may serve as a basis for the creation of a healthy working environment wherein coping strategies are enhanced. Nurse administrators and educators may develop a plan that enhances nurse awareness of mechanisms for chronic stress reduction and coping behaviours.

## AUTHOR CONTRIBUTIONS

AK, EK, AGA and PO were involved in the conceptualization, study design, the data collection, data analysis and presentation. BA, NH and VN supervised the work, screening and validity. All authors were involved in the writing, review and finalization of the manuscript.

## FUNDING INFORMATION

The authors received no funding for this research.

## CONFLICT OF INTEREST STATEMENT

The authors declared no potential conflict of interests concerning the research, and or publication of the article.

## ETHICS STATEMENT

Institutional review board approval proceeded with the distribution of the self‐administered questionnaire. Written informed consent was required for the recruitment of the study participants. The confidentiality and privacy of the participants were upheld throughout the whole research procedure process. Participants had the option to withdraw at any moment from the study. Egerton University Institutional review board approval (EG‐2022‐178).

## Data Availability

Data are available from the corresponding author on reasonable request.

## References

[nop21831-bib-0001] Ayalew, E. , Workineh, Y. , Semachew, A. , Woldgiorgies, T. , Kerie, S. , Gedamu, H. , & Zeleke, B. (2021). Nurses' intention to leave their job in sub‐Saharan Africa: A systematic review and meta‐analysis. Heliyon, 7(6), e07382. 34258453 10.1016/j.heliyon.2021.e07382 PMC8253915

[nop21831-bib-0002] Babapour, A.‐R. , Gahassab‐Mozaffari, N. , & Fathnezhad‐Kazemi, A. (2022). Nurses' job stress and its impact on quality of life and caring behaviors: A cross‐sectional study. BMC Nursing, 21(1), 1–10. 35361204 10.1186/s12912-022-00852-y PMC8968092

[nop21831-bib-0003] Bahrami, M. (2016). Nurses' quality of life in medical‐surgical wards of an oncology center affiliated to the Isfahan University of Medical Sciences. Nursing Journal of the Vulnerable, 3(7), 36–46.

[nop21831-bib-0004] Betke, K. , Basińska, M. A. , & Andruszkiewicz, A. (2021). Sense of coherence and strategies for coping with stress among nurses. BMC Nursing, 20(1), 1–10. 34162376 10.1186/s12912-021-00631-1 PMC8220127

[nop21831-bib-0005] Ceccato, S. , Kettner, S. E. , Kudielka, B. M. , Schwieren, C. , & Voss, A. (2018). Social preferences under chronic stress. PLoS One, 13(7), e0199528. 30020926 10.1371/journal.pone.0199528 PMC6051590

[nop21831-bib-0006] Clark, S. , Cotton, C. , Margolis, R. , & Kohler, H. (2020). The psychological benefits of marriage and children in rural Malawi. Studies in Family Planning, 51(3), 251–272. 32720321 10.1111/sifp.12133 PMC7778373

[nop21831-bib-0007] De Diego‐Cordero, R. , Iglesias‐Romo, M. , Badanta, B. , Lucchetti, G. , & Vega‐Escaño, J. (2022). Burnout and spirituality among nurses: A scoping review. Explore, 18(5), 612–620. 34429263 10.1016/j.explore.2021.08.001

[nop21831-bib-0008] Ejebu, O.‐Z. , Dall'Ora, C. , & Griffiths, P. (2021). Nurses' experiences and preferences around shift patterns: A scoping review. PLoS One, 16(8), e0256300. 34398904 10.1371/journal.pone.0256300 PMC8367008

[nop21831-bib-0009] Garrosa, E. , Moreno‐Jiménez, B. , Rodríguez‐Muñoz, A. , & Rodríguez‐Carvajal, R. (2011). Role stress and personal resources in nursing: A cross‐sectional study of burnout and engagement. International Journal of Nursing Studies, 48(4), 479–489. 20828694 10.1016/j.ijnurstu.2010.08.004

[nop21831-bib-0010] Gavin, B. , Hayden, J. , Adamis, D. , & McNicholas, F. (2020). Caring for the psychological well‐being of healthcare professionals in the Covid‐19 pandemic crisis. Irish Medical Journal, 113(4), 51. 32268045

[nop21831-bib-0011] Grundström, J. , Konttinen, H. , Berg, N. , & Kiviruusu, O. (2021). Associations between relationship status and mental well‐being in different life phases from young to middle adulthood. SSM‐Population Health, 14, 100774. 33869721 10.1016/j.ssmph.2021.100774 PMC8040327

[nop21831-bib-0012] Heuel, L. , Lübstorf, S. , Otto, A.‐K. , & Wollesen, B. (2022). Chronic stress, behavioral tendencies, and determinants of health behaviors in nurses: A mixed‐methods approach. BMC Public Health, 22(1), 1–13. 35354449 10.1186/s12889-022-12993-5 PMC8967083

[nop21831-bib-0013] Jacoby, R. , Greenfeld Barsky, K. , Porat, T. , Harel, S. , Hanalis Miller, T. , & Goldzweig, G. (2021). Individual stress response patterns: Preliminary findings and possible implications. PLoS One, 16(8), e0255889. 34388203 10.1371/journal.pone.0255889 PMC8363008

[nop21831-bib-0014] Kersting, C. , Zimmer, L. , Thielmann, A. , & Weltermann, B. (2019). Chronic stress, work‐related daily challenges and medicolegal investigations: A cross‐sectional study among German general practitioners. BMC Family Practice, 20(1), 1–8. 31651239 10.1186/s12875-019-1032-6 PMC6813962

[nop21831-bib-0015] Lazarus, R. S. , & Folkman, S. (1987). Transactional theory and research on emotions and coping. European Journal of Personality, 1(3), 141–169.

[nop21831-bib-0016] Li, Y. , Wang, Y. , Lv, X. , Li, R. , Guan, X. , Li, L. , Li, J. , & Cao, Y. (2022). Effects of factors related to shift work on depression and anxiety in nurses. Frontiers in Public Health, 10(12), 721–731. 10.3389/fpubh.2022.926988 PMC9326492 35910870

[nop21831-bib-0017] Lim, J. , Bogossian, F. , & Ahern, K. (2010). Stress and coping in Australian nurses: A systematic review. International Nursing Review, 57(1), 22–31. 20487471 10.1111/j.1466-7657.2009.00765.x

[nop21831-bib-0018] Long, K. N. G. , Chen, Y. , Potts, M. , Hanson, J. , & VanderWeele, T. J. (2020). Spiritually motivated self‐forgiveness and divine forgiveness, and subsequent health and well‐being among middle‐aged female nurses: An outcome‐wide longitudinal approach. Frontiers in Psychology, 11, 1337. 32733311 10.3389/fpsyg.2020.01337 PMC7363844

[nop21831-bib-0019] López‐Cabarcos, M. Á. , Vázquez‐Rodríguez, P. , & Quiñoá‐Piñeiro, L. M. (2022). An approach to employees' job performance through work environmental variables and leadership behaviours. Journal of Business Research, 140, 361–369.

[nop21831-bib-0020] Martinez, V. M. L. (2021). The importance of workplace exercise/A importancia da ginastica laboral. Revista Brasileira de Medicina Do Trabalho, 19(4), 523–529. 35733538 10.47626/1679-4435-2021-666 PMC9162294

[nop21831-bib-0021] Musiimenta, P. , Munezero, J. B. T. , Moazzam, M. L. , & Kabanyoro, A. (2022). Occupational stress and its health outcomes among nurses in health facilities of Bushenyi district Western Uganda. Special Journal of Public Health, Nutrition, and Dietetics, 2(2), 1–12.

[nop21831-bib-0022] Nabirye, R. C. , Brown, K. C. , Pryor, E. R. , & Maples, E. H. (2011). Occupational stress, job satisfaction and job performance among hospital nurses in Kampala, Uganda. Journal of Nursing Management, 19(6), 760–768. 21899629 10.1111/j.1365-2834.2011.01240.x

[nop21831-bib-0023] Nadeem, F. , Sadiq, A. , Raziq, A. , Iqbal, Q. , Haider, S. , Saleem, F. , & Bashaar, M. (2021). Depression, anxiety, and stress among nurses during the COVID‐19 Wave III: Results of a cross‐sectional assessment. Journal of Multidisciplinary Healthcare, 14, 3093–3101. 34785902 10.2147/JMDH.S338104 PMC8580291

[nop21831-bib-0024] Odigie, A. (2016). Stress Management for Healthcare Professionals. Yrkeshögskolan Arcada.

[nop21831-bib-0025] Perelli‐Harris, B. , Hoherz, S. , Addo, F. , Lappegård, T. , Evans, A. , Sassler, S. , & Styrc, M. (2018). Do marriage and cohabitation provide benefits to health in mid‐life? The role of childhood selection mechanisms and partnership characteristics across countries. Population Research and Policy Review, 37(5), 703–728. 30546176 10.1007/s11113-018-9467-3 PMC6267248

[nop21831-bib-0026] Perera, C. K. , Pandey, R. , & Srivastava, A. K. (2018). Role of religion and spirituality in stress management among nurses. Psychological Studies, 63(2), 187–199.

[nop21831-bib-0027] Rosenberg, K. (2020). Shift work adversely affects mental health. The American Journal of Nursing, 120(3), 67. 10.1097/01.NAJ.0000656368.67026.9f 32079804

[nop21831-bib-0028] Santana, I. R. , Montes, M. A. , Chalkley, M. , Jacobs, R. , Kowalski, T. , & Suter, J. (2020). The impact of extending nurse working hours on staff sickness absence: Evidence from a large mental health hospital in England. International Journal of Nursing Studies, 112, 103611. 32451063 10.1016/j.ijnurstu.2020.103611 PMC7700891

[nop21831-bib-0029] Shah, C. H. , & Brown, J. D. (2020). Reliability and validity of the Short‐Form 12 Item Version 2 (SF−12v2) health‐related quality of life survey and disutilities associated with relevant conditions in the US older adult population. Journal of Clinical Medicine, 9(3), 661. 32121371 10.3390/jcm9030661 PMC7141358

[nop21831-bib-0030] Shiden, T. , Chubike, N. E. , & William, O. (2017). Workplace stress induced among nurses at Mbarara Regional Referral Hospital, south West Uganda. Journal of Nursing Care, 6, 424.

[nop21831-bib-0032] Sweeney, E. , Cui, Y. , Yu, Z. M. , Dummer, T. J. B. , DeClercq, V. , Forbes, C. , Grandy, S. A. , Keats, M. R. , & Adisesh, A. (2021). The association between mental health and shift work: Findings from the Atlantic PATH study. Preventive Medicine, 150, 106697. 34175348 10.1016/j.ypmed.2021.106697

[nop21831-bib-0033] Wollesen, B. , Hagemann, D. , Pabst, K. , Schlüter, R. , Bischoff, L. L. , Otto, A.‐K. , Hold, C. , & Fenger, A. (2019). Identifying individual stressors in geriatric nursing staff—A cross‐sectional study. International Journal of Environmental Research and Public Health, 16(19), 3587. 31557867 10.3390/ijerph16193587 PMC6802224

[nop21831-bib-0034] Yamaoka, K. (2008). Social capital and health and well‐being in East Asia: A population‐based study. Social Science & Medicine, 66(4), 885–899. 18158206 10.1016/j.socscimed.2007.10.024

